# Effects of precursor solution composition on the performance and I-V hysteresis of perovskite solar cells based on CH_3_NH_3_PbI_3-x_Cl_x_

**DOI:** 10.1186/s11671-017-1872-8

**Published:** 2017-02-03

**Authors:** Z. L. Zhang, B. Q. Men, Y. F. Liu, H. P. Gao, Y. L. Mao

**Affiliations:** 1 School of Physics and Electronics, Henan University, Kaifeng, 475004 China; 2 Henan Vocational College of Agriculture, Zhongmu, 451450 China; 3 Institute for Computational Materials Science, Henan University, Kaifeng, 475004 China

**Keywords:** I-V hysteresis, Precursor solution composition, CH_3_NH_3_PbI_3-x_Cl_x_

## Abstract

Precursor solution of CH_3_NH_3_PbI_3-x_Cl_x_ for perovskite solar cells was conventionally prepared by mixing PbCl_2_ and CH_3_NH_3_I with a mole ratio of 1:3 (PbCl_2_:CH_3_NH_3_I). While in the present study, CH_3_NH_3_PbI_3-x_Cl_x_-based solar cells were fabricated using the precursor solutions containing PbCl_2_ and CH_3_NH_3_I with the mole ratios of 1:3, 1.05:3, 1.1:3, and 1.15:3, respectively. The results display that the solar cells with the mole ratio of 1.1:3 present higher power conversion efficiency and less I-V hysteresis than those with the mole ratio of 1:3. Based on some investigations, it is concluded that the higher efficiency could be due to the smooth and pinhole free film formation, high optical absorption, suitable energy band gap, and the large electron transfer efficiency, and the less I-V hysteresis may be attributed to the small low frequency capacitance of the device.

## Background

Organometal halide perovskite solar cells (PSCs) have attracted much attention over the last several years due to their outstanding properties, such as large absorption coefficient, high electron-hole diffusion length, and high charge carrier mobility [1–6]. The power conversion efficiency (PCE) has increased from 3.8 to 22% [7]. The typical architectures of PSCs mainly contain electron transporting layer (ETL)/perovskite/hole transporting layer (HTL) (n-i-p) and HTL/perovskite/ETL (p-i-n) structures [8]. In the CH_3_NH_3_PbX_3_ (X = I, Br, Cl) family, a mixed halide perovskite CH_3_NH_3_PbI_3-x_Cl_x_ (MAPbI_3-x_Cl_x_) has been proved a large diffusion length (~1 μm), which could be applied for planar heterojunction solar cells with improved device performance [9, 10]. Some groups have reported the results of the MAPbI_3-x_Cl_x_-based solar cells [11–13], in which the highest PCE is 19.3% [14].

The precursor solution of MAPbI_3-x_Cl_x_ is conventionally prepared by mixing PbCl_2_ and CH_3_NH_3_I with a mole ratio of 1:3 (PbCl_2_:CH_3_NH_3_I). While there was no or only trace amount of Cl to be detected [15, 16]. Some studies have been performed to investigate the role of Cl in the MAPbI_3-x_Cl_x_ film formation [17, 18]. A widely accepted opinion is that Cl ion in organometal halide perovskite can boost the mobility of excitons and the charge carrier transport [19–21]. A few groups have fabricated MAPbI_3-x_Cl_x_ solar cells using the precursor solutions containing excess PbCl_2_ to investigate its effect on the performance of solar cells based on the I-V measurement with single scan direction [18, 21–23]. It has been reported that hysteretic effects were observed during the I-V measurement of the perovskite solar cells [24]. I-V hysteresis could lead to an over- or underestimation of the PCE if it is not considered. Up to now, there are few reports to investigate the effects of excess PbCl_2_ on the PCE and I-V hysteresis of MAPbI_3-x_Cl_x_ solar cells by considering the hysteretic effect.

Therefore in the present study, MAPbI_3-x_Cl_x_-based solar cells were fabricated using the precursor solutions containing different mole ratios of PbCl_2,_ and CH_3_NH_3_I. I-V measurements were carried out with reverse scan (RS) and forward scan (FS). The photovoltaic parameters were obtained from the I-V curves averaged with RS and FS. Based on the measurements, the effects of excess PbCl_2_ on the PCE and the I-V hysteresis of the solar cells were investigated. One of the novelties of this work is that the photovoltaic parameters were obtained by an average of RS and FS to improve the accuracy of data. The other is the observation and investigation of the effect of excess PbCl_2_ on I-V hysteresis.

## Methods

### Materials preparation

Methylammonium iodide (CH_3_NH_3_I) was synthesized with a method reported in the literature [25]. The perovskite precursor solutions (40 wt%) were obtained by mixing PbCl_2_ and CH_3_NH_3_I (MAI) in anhydrous *N*,*N*-Dimethylformamide (DMF) at 60 °C with the mole ratios of 1:3, 1.05:3, 1.1:3, and 1.15:3 (PbCl_2_ to MAI), respectively.

### Solar cell fabrication

Perovskite solar cells with a structure of n-i-p were fabricated. FTO-coated glass substrate (~15 ohm/sq, NPG, Japan) was patterned and cleaned with detergent, acetone, 2-propanol, and ethanol for 15 min by sonication. Then the substrate was treated by oxygen plasma for 20 min. A hole-blocking layer of compact TiO_2_ was deposited by spin-coating, a mildly acidic solution of titanium isopropoxide (Aladdin reagent) in ethanol (350 μl in 5 ml ethanol with 0.013 M HCl) at 2000 rpm for 30 s and annealed at 500 °C for 30 min. A mesoporous TiO_2_ layer composed of commercial TiO_2_ paste (Dyesol 18NRT, Dyesol) diluted in ethanol (1:3.5, weight ratio) was then deposited on the top of compact layer by spin-coating at 5000 rpm for 30 s. After drying at 125 °C, the TiO_2_ films were annealed at 500 °C for 30 min. The perovskite precursor solution was spin-coated on the mesoporous TiO_2_ film at 2000 rpm for 45 s in an argon-filled glove box. The sample was dried on a hotplate for 60 min at 110 °C. The hole-transporter layer was formed by spincoating a spiro-OMeTAD solution at 2000 rpm for 45 s. Finally, a gold layer with the thickness of 80 nm was deposited on top of the device by thermal evaporation in air.

### Characterization

X-ray diffraction (XRD) patterns were carried out on a DX-2700 diffractometer. UV-vis absorption spectra were performed on a UV–vis spectrophotometer (Varian Cary 5000). Morphologies and microstructures were obtained by a scanning electron microscope (SEM, JEM-7001 F, JEOL). Photocurrent-voltage (I-V) curves were carried out with a Keithley 2440 Sourcemeter under AM 1.5 G illumination with 100-mW/cm^2^ intensity from a Newport Oriel Solar Simulator. The active area of the device was 0.1 cm^2^ determined with a mask. Steady-state photoluminescence (PL) and time-resolved photoluminescence (TRPL) spectra were collected using a fluorometer (FLS 980E, Edinburgh Photonics). Capacitance-frequency measurements were performed under a forward bias of 0.6 V under 1 sun illumination conditions using an electrochemical workstation (RST5200, Zhengzhou Shiruisi Instrument Co., Ltd.) with the frequency range from 0.1 to 1000 Hz. The electrochemical impedance (IS) measurements were carried out with an electrochemical workstation (CHI660e, Shanghai CHI Co., Ltd.) in the frequency range from 0.1 to 100 kHz, in which an alternative signal with 5 mV magnitude was applied.

## Results and discussion

**Fig. 1:**
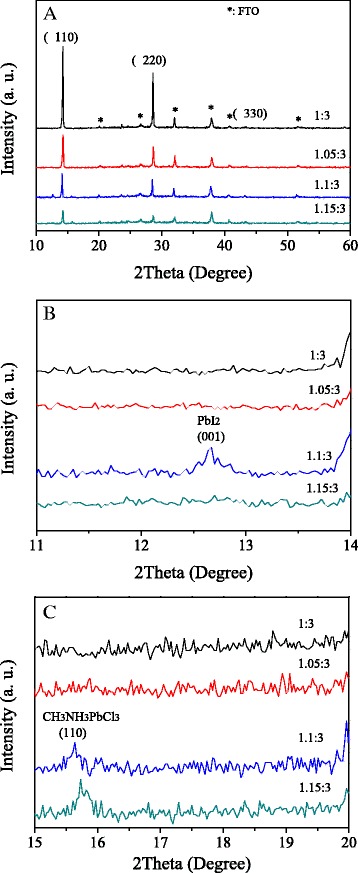
XRD patterns of MAPbI_3-x_Cl_x_ films with different mole ratios at the range of (**a**) 10-60 degree, (**b**) 11-14 degree, and (**c**) 15-20 degree

Figure 1 shows the XRD patterns of MAPbI_3-x_Cl_x_ films with different mole ratios. Three main diffraction peaks at about 14.2°, 28.6°, and 43.1° are ascribed to (110), (220), and (330) lattice planes of halide perovskite with a tetragonal structure [26]. This indicates that the perovskite films with tetragonal structure are formed. A weak peak located at 12.7° for the sample with the ratio of 1.1:3 (Fig. 1b) can be assigned to the (001) diffraction peak of PbI_2_ [10]. The peak at about 15.6° for the samples with the mole ratios of 1.1:3 and 1.15:3 (Fig. 1c) can be assigned to the (110) diffraction peak of CH_3_NH_3_PbCl_3_ [18]. This agrees with the previous reports that Cl incorporation in an iodide-based structure was only at low concentration, and phase separation readily occurred with increased concentration [22, 27]. The PbI_2_ phase appeared for the sample with the mole ratio of 1.1:3 and then disappeared for that with the mole ratio of 1.15:3 (Fig. 1b). While the CH_3_NH_3_PbCl_3_ phase appeared in both the samples with the mole ratios of 1.1:3 and 1.15:3 (Fig. 1c). This is in accord with the reported MAPbI_3-x_Cl_x_ film growth process [23]. As a nucleation center, PbCl_2_ induces the nucleation of PbI_2_. PbI_2_ acts with MAI to form the perovskite film and exhausts the available Pb ions to form CH_3_NH_3_PbCl_3_.

**Fig. 2:**
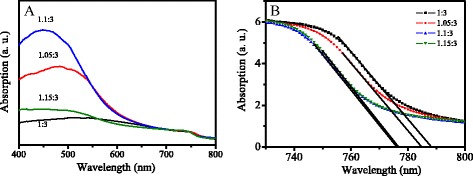
UV-vis absorption spectra of MAPbI_3-x_Cl_x_ films with different mole ratios at the range of (**a**) 400-800 nm, and (**b**) 730-800 nm

Figure 2a shows the UV-vis absorption spectra of MAPbI_3-x_Cl_x_ films with different mole ratios. The absorption intensity increases firstly, and then decreases with the increase of mole ratio, which is the strongest at the mole ratio of 1.1:3. Figure 2b shows the absorption spectra of MAPbI_3-x_Cl_x_ films at the range from 730 to 800 nm. The absorption edge is obtained by extrapolating from the absorption of direct transition [28]. The band gap of MAPbI_3-x_Cl_x_ can be estimated from the absorption edge to be 1.573, 1.580, 1.598, and 1.596 eV for MAPbI_3-x_Cl_x_ with the mole ratio of 1:3, 1.05:3, 1.1:3, and 1.15:3, respectively.

**Fig. 3:**
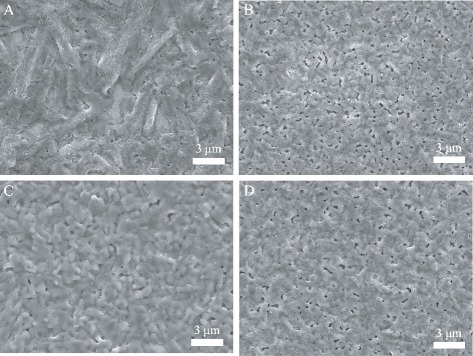
SEM images of the MAPbI_3-x_Cl_x_ films with the mole ratio of (**a**) 1:3, (**b**) 1.05:3, (**c**) 1.1:3, and (**d**) 1.15:3

Figure 3 shows the SEM images of the MAPbI_3-x_Cl_x_ films with different mole ratios. It is observed that some needle-like crystals for the film with the mole ratio of 1:3 (Fig. 3a). The films with the mole ratios of 1.05:3 and 1.15:3 become smooth and cover all the substrate with some small pinholes (Fig. 3b, d). At the mole ratio of 1.1:3, the pinholes disappeared and the substrate was fully covered by the MAPbI_3-x_Cl_x_ film (Fig. 3c). According to the previous reports [23, 29], PbCl_2_ colloids in the precursor solution act as heterogeneous nucleation sites for the perovskite film formation. When excess PbCl_2_ was introduced, the heterogeneous nucleation sites increased rationally, which possibly enhanced the morphology eventually. As the amount of PbCl_2_ further increased, the grain size of the perovskite film slightly increased, and the surface becomes mother.

**Fig. 4:**
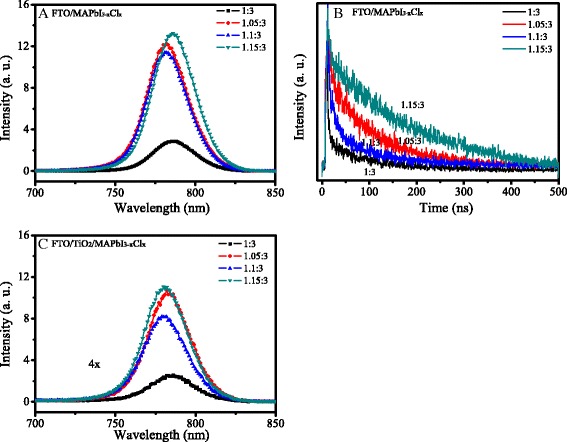
**a** PL and **b** TRPL spectra of FTO/MAPbI_3-x_Cl_x_ samples. **c** PL spectra of FTO/TiO_2_/MAPbI_3-x_Cl_x_ samples

Figure 4a shows the PL spectra of MAPbI_3-x_Cl_x_ films with different mole ratios on FTO substrate. The peak at ~780 nm could be from the emission of MAPbI_3-x_Cl_x_ [9]. The PL intensities of the films with the mole ratios of 1.05:3, 1.1:3, and 1.15:3 are higher than that of 1:3. The TRPL spectra of MAPbI_3-x_Cl_x_ films with different mole ratios on FTO substrate are shown in Fig. 4b. The TRPL curve was fitted with an exponential diffusion model, and the exciton lifetime is 58, 79, 67, and 177 ns for the perovskite film with the mole ratio of 1:3, 1.05:3, 1.1:3, and 1.15:3, respectively. The exciton lifetimes of the films with the mole ratio of 1.05:3, 1.1:3, and 1.15:3 are longer than that of that of 1:3. The higher PL intensities could be due to their longer exciton lifetimes [30, 31]. The enhanced exciton lifetime indicates the reduced recombination in the MAPbI_3-x_Cl_x_ films. To investigate the charge transfer between MAPbI_3-x_Cl_x_ film and TiO_2_, the PL spectra of FTO/TiO_2_/MAPbI_3-x_Cl_x_ samples were performed and shown in Fig. 4c. Compared with the PL spectra of FTO/MAPbI_3-x_Cl_x_ (Fig. 4a), there is a quenching effect when the MAPbI_3-x_Cl_x_ layer contacts with TiO_2_ film, which is due to the electron injection from MAPbI_3-x_Cl_x_ to TiO_2_. The charge transfer efficiency can be estimated by the PL intensity ratio of FTO/TiO_2_/MAPbI_3-x_Cl_x_ to FTO/MAPbI_3-x_Cl_x_, which is 0.25, 0.21, 0.19, and 0.24 for the perovskite films with the mole ratio of 1:3, 1.05:3, 1.1:3, and 1.15:3, respectively. The PL intensity ratio of the sample with the mole ratio of 1.1:3 is smaller than the others, which indicates a more efficient electron transfer to TiO_2_. This could be due to a stronger interfacial coupling at the interface [32].

**Fig. 5:**
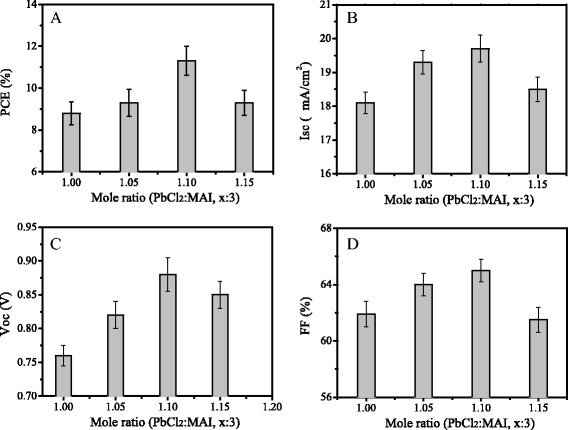
Photovoltaic parameters of the solar cells using the precursor solutions with different mole ratios. **a** Isc, **b** Voc, **c** FF, and **d** PCE. The data were obtained from 20 pieces of devices for each of precursor solutions

Perovskite solar cells were fabricated using the precursor solutions with different mole ratios with a structure of FTO/c-TiO_2_/mp-TiO_2_/MAPbI_3-x_Cl_x_/spiro-oMeTAD/Au. Figure 5 shows the photovoltaic parameters of the solar cells, which were obtained from 20 pieces of devices for each of precursor solutions. The short current (*J*_sc_), open voltage (*V*_oc_), fill factor (FF) and power conversion efficiency (PCE) were obtained from I-V curves averaged with reverse scan (RS) and forward scan (FS). These parameters are listed in Table 1. With the increase of mole ratio, the parameters of the solar cells were firstly increased, and then decreased. The solar cells with the mole ratio of 1.1:3 present an enhanced performance. Compared with those of solar cells with the mole ratio of 1:3, the *V*_oc_, *J*_sc_, FF, and PCE of the solar cells with the mole ratio of 1.1:3 were increased to 0.88 V, 19.7 mA/cm^2^, 65%, and 11.3% from 0.76 V, 18.1 mA/cm^2^, 61.9%, and 8.8%, respectively.

**Table 1:** Photovoltaic parameters of perovskite solar cells as a function of different mole ratios of PbCl_2_ and MAI

PbCl_2_:MAI	*V*_oc_ (V)	*J*_sc_ (mA/cm^2^)	FF (%)	*η* (%)
1:3	0.76 ± 0.01	18.1 ± 0.2	61.9 ± 1.6	8.8 ± 0.1
1.05:3	0.82 ± 0.02	19.3 ± 0.3	64.0 ± 1.5	9.3 ± 0.2
1.1:3	0.88 ± 0.01	19.7 ± 0.1	65.0 ± 0.5	11.3 ± 0.2
1.15:3	0.85 ± 0.01	18.5 ± 0.2	61.5 ± 1.5	9.3 ± 0.3

**Fig. 6:**
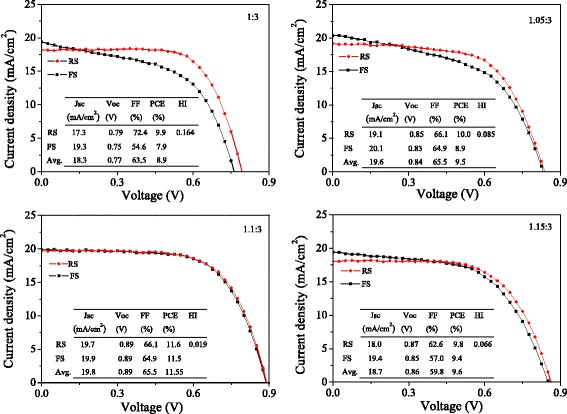
Current density-voltage (I-V) curves of the best solar cells using the precursor solutions with different mole ratios

Figure 6 shows the current density-voltage (I-V) curves of the best solar cells using the precursor solutions with different mole ratios. It was found that the degree of I-V hysteresis depends on the precursor composition. This phenomenon was always observed in our experiments. I-V hysteresis index (HI) is defined by the following equation [33],

$$ \mathrm{hysteresis}\;\mathrm{index}=\frac{J_{\mathrm{RS}}\left(0.8{V}_{\mathrm{oc}}\right)-{J}_{\mathrm{FS}}\left(0.8{V}_{\mathrm{oc}}\right)}{J_{\mathrm{RS}}\left(0.8{V}_{\mathrm{oc}}\right)} $$

where *J*_RS_(0.8*V*_oc_) and *J*_FS_(0.8*V*_oc_) stand for the photocurrent density at 80% of *V*_oc_ for the RS and FS, respectively. The calculated hysteresis index values are 0.164, 0.085, 0.019, and 0.066 for the I-V curves with the mole ratio of 1:3, 1.05:3, 1.1:3, and 1.15:3, respectively. With the increase of mole ratio, the hysteresis degree first decreases, and then increases. At the mole ratio of 1.1:3, the hysteresis index value is the smallest. The high PCE of 11.55% with less I-V hysteresis was obtained using the precursor solution with the mole ratio of 1.1:3.

**Fig. 7:**
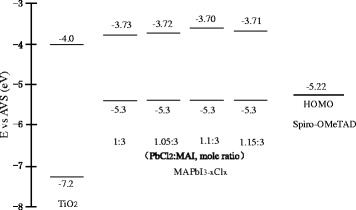
Schematic of the energy band diagrams of TiO_2_, MAPbI_3-x_Cl_x_, and Spiro-OMeTAD.

To get an insight into the enhanced performance and less I-V hysteresis of the solar cells with the mole ratio of 1.1:3, some investigations were performed. Based on the energy band gaps calculated from the absorption spectra (Fig. 2) and the literature [33], the energy band diagrams of TiO_2_, MAPbI_3-x_Cl_x_, and Spiro-OMeTAD are shown in Fig. 7. The conduction band offset between MAPbI_3-x_Cl_x_ and TiO_2_ is the largest for the mole ratio of 1.1:3 due to its wide band gap, which might be one of the reasons to present a higher voltage [28]. Moreover, the larger conduction band offset might contribute to its increased current density, because the band offset has been proved to be a driving force for charge transfer between conduction bands in the heterojunction [34, 35]. This speculation was confirmed by the photoluminescence (PL) results.

**Fig. 8:**
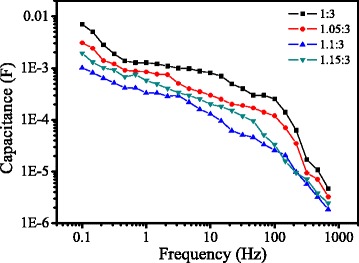
Capacitance-frequency plots of the solar cells directly measured from an electrochemical workstation

Hysteretic effects during I-V measurements have been observed in perovskite solar cells. It has been proposed that the slow decay process of the capacitive charging or discharging current during voltage sweep induces the non-steady state photocurrent and I-V hysteresis [33, 36, 37]. The non-steady state photocurrents could be due to the capacitance at low frequency (0.1 ~ 1 Hz) resulting from electrode polarization at perovskite/electrode interfaces [33]. To understand the I-V hysteresis of the solar cells with different precursor compositions, the capacitance of the devices were directly measured with an electrochemical workstation. Figure 8 shows the dependence of capacitance on frequency. The low frequency capacitance (*C*_LF_) is observed near 10^−1^ Hz. With the increase of the mole ratio, *C*_LF_ decreases firstly, and then increases, which is the smallest at the mole ratio of 1.1:3. The smaller *C*_LF_ indicates the less polarization which could be the origin of the I-V hysteresis [33]. The variation of *C*_LF_ with the mole ratio agrees with the I-V hysteresis tendency shown in Fig. 4.

**Fig. 9:**
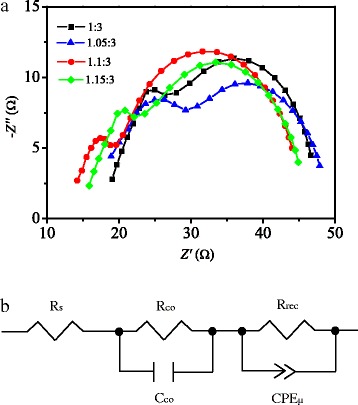
**a** Typical Nyquist plots for the perovskite solar cells. The simulation result (*solid line*) is fitted to experimental data (*symbols*). **b** Equivalent circuit applied to fit the Nyquist plots

To investigate the reason of capacitance decrease, the impedance spectra of the solar cells were measured. Figure 9a shows the Nyquist plots of the cells based on the different mole ratios, in which the symbols are the experimental data and the solid lines are the fitting results. There are two RC arcs contained in the plots. Figure 9b shows the equivalent circuit used to fit the data. The high-frequency RC element could be ascribed to the contact resistance (*R*_co_) at the interfaces, while the low-frequency element may be attributed to the recombination resistance (*R*_rec_) and chemical capacitance (*C*_μ_) of the device, and the *R*_s_ is a series resistance [38]. The parameters obtained by fitting are listed in Table 2. The *R*_co_ (10.6 Ω) of solar cells based on precursor solution with mole ratio of 1.1:3 is smaller than that of the other precursor solution. This indicates that the perovskite film with the mole ratio of 1.1:3 provides better contact with electron transporting layer and hole transporting layer than the other perovskite film. Thus, the decreased capacitance of the solar cells with the mole ratio of 1.1:3 could be due to the better contact of perovskite film with ETL and HTL [39].

**Table 2:** Fitting parameters for EIS data

Sample	*R*_s_/Ω	*R*_co_/Ω	*R*_rec_/Ω	*C*_co_/F	CPE-T/F
1:3	17.7	21.0	23.7	1.5E-7	3.6E-6
1.05:3	17.1	17.8	27.5	1.6E-8	2.5E-6
1.1:3	13.5	10.6	28.5	1.2E-7	6.9E-7
1.15:3	15.2	16.8	27.6	1.3E-7	5.1E-6

## Conclusion

The solar cells based on MAPbI_3-x_Cl_x_ were fabricated using the precursor solutions containing the mole ratio of 1:3, 1.05:3, 1.1:3, and 1.15:3. I-V curves were obtained by both reverse scan and forward scan, from which the photovoltaic parameters were calculated by taking the average of them. The results displayed that the solar cells with the mole ratio of 1.1:3 present higher PCE and less I-V hysteresis. To get an insight into the results, some investigations were performed. The higher PCE could be due to the smooth and pinhole-free film formation, high optical absorption, suitable energy band gap, and the large electron transfer efficiency. The less I-V hysteresis may be attributed to the small low frequency capacitance of the device.
